# Jaak Panksepp’s primary emotions are associated with Diener’s global life satisfaction: How low arousal of the SADNESS/separation distress system could form the core of life satisfaction?

**DOI:** 10.1017/pen.2024.4

**Published:** 2024-11-04

**Authors:** Kenneth L. Davis, Christian Montag

**Affiliations:** 1 Pegasus International, Greensboro, NC, 27408, USA; 2 Department of Molecular Psychology, Institute of Psychology and Education, Ulm University, Ulm, BW, 89081, Germany

**Keywords:** emotions, life satisfaction, Panksepp, primary emotional systems, well-being

## Abstract

We compared Ed Diener’s *Satisfaction With Life Scale* (SWLS), which was designed as a purely cognitive measure of global life satisfaction, with the *Affective Neuroscience Personality Scales 3.1*, which provides self-report measures of Panksepp’s six primary emotions (excluding LUST), in two English-speaking samples: a main sample and a hold-out validation sample. Our data showed robust negative correlations between higher satisfaction with life and lower FEAR, lower SADNESS/Separation Distress, and positive associations (albeit less strong) between higher satisfaction with life and higher PLAY and SEEKING in both samples. The relationships between the SWLS and at least four of Panksepp’s primary emotions suggest Diener’s SWLS is not purely cognitive and includes a strong affective component. In addition, detailed analysis of the negative correlation between the SWLS and the ANPS 3.1 SADNESS scale provides insight into the importance of the low arousal end of the SADNESS/Separation Distress brain system and supports the idea of a continuum of psychological states from high SADNESS including loneliness and depression to low SADNESS psychological states characterized by social comfort, self-confidence, and social strength.

## 1. Introduction

This research article explores new evidence that Jaak Panksepp’s primary-process GRIEF/SADNESS/Separation Distress brain system (Panksepp, 1998; Panksepp & Watt, 2011) is subjectively experienced as a broad affective spectrum that extends far beyond its narrower and more typical representation as a painfully experienced emotion with strong links to depression (Montag et al., 2017; Montag, Sanwald, et al., 2022). The impetus for this broader discussion of the SADNESS/Separation Distress brain system comes from data reported in this paper, which highlights positive emotional states correlated with low expression of the SADNESS system (measured as a trait). In other words, we are talking about the less discussed pleasant feelings experienced on the opposite side of the SADNESS system’s social affective spectrum linked with unpleasant social rejection, social loss, and depression. That is, when the powerful SADNESS system is not triggered by the likes of separation distress, and by contrast, is responding to the experience of social connection, we experience pleasing feelings at the opposite end of the SADNESS system’s affective spectrum. Although most attention has been given to the affective emotions experienced when Jaak Panksepp’s primary brain systems are aroused (typically using local brain stimulation or social isolation in the case of GRIEF/SADNESS; Panksepp, 1998), it may be equally important to understand the affects experienced when these brain systems are not aroused. Indeed, what less studied subjective states are enabled in these low arousal conditions? As we report here, the Affective Neuroscience Personality Scales 3.1 SADNESS scale (Montag, Elhai, & Davis, 2021) correlated approximately as highly (although negatively) with ratings of personal life satisfaction (Diener et al., 1985), as it has positively with measures of depression in the past (Montag et al., 2017).

### 1.1 Comparing the affective neuroscience personality scales (here ANPS 3.1) and Diener’s satisfaction with life scale (SWLS)

In 1984 Ed Diener published an article in *Psychological Bulletin*, entitled “Subjective Well-Being” (Diener, 1984) effectively launching subjective well-being as a research topic. Then, in 1985, his group at the University of Illinois published the “Satisfaction With Life Scale” (Diener et al., 1985). Diener concluded that subjective well-being consisted of three components: positive affect, negative affect, and life satisfaction. He conceived life satisfaction as the cognitive-judgmental component of well-being and used factor analysis of items reflecting global life satisfaction as well as both Positive Affect and Negative Affect to identify “cognitive” life satisfaction items that loaded on a single factor independent of Positive Affect and Negative Affect factors. From the 10 items that loaded on his cognitive judgment scale, he selected the best five for the final “Satisfaction With Life Scale” (Diener et al., 1985), which continues to be used (Emerson et al., 2017).

Interestingly, Pavot and Diener (1993) acknowledged that the “cognitive” global life satisfaction scale positively correlated with Extraversion and negatively with Neuroticism (reverse of Emotional Stability), which from Jaak Panksepp’s Affective Neuroscience viewpoint would both be considered as emotional affects (here persons being more prone to experience positive or negative emotionality). For insights from a large-scale study on life satisfaction and personality see a work from a few years ago (Lachmann et al., 2018). A meta-analysis on associations between Panksepp’s primary emotional systems and the Big Five personality scales revealed that Extraversion is closely related to the primary PLAY emotion and Emotional Stability is closely related to low SADNESS, FEAR, and ANGER (Marengo et al., 2021). So, from an Affective Neuroscience perspective, despite Diener’s factor analysis separating the SWLS from emotional affects, SWLS has never been a purely cognitive measure.

Indeed, from an Affective Neuroscience perspective, one would consider an SWLS item such as “I am satisfied with my life” to communicate an affective “valence,” which would qualify it as an evaluative emotional judgment. But the more basic underlying conceptual question may be whether it is even possible for individuals to make purely cognitive subjective self-report judgments about their lives without evaluating the emotional affective nature of their life experiences?

### 1.2 Research objectives

Against the background of the so far put forward discussions in the literature, we investigate in the present paper for the first time the associations between the Affective Neuroscience Personality Scales (3.1.) as a self-report measure for Panksepp’s primary emotional systems and global life satisfaction as measured by Ed Diener’s scale. In light of the literature, we hypothesize that the SWLS would correlate positively with the SEEKING, CARE, and PLAY emotions and correlate negatively with the ANGER, FEAR, and SADNESS emotions. Our study also helps draw attention to the low arousal side of the Affective Neuroscience SADNESS as well as FEAR brain systems. The affectively positive nature of Diener’s SWLS scale helps us focus on the ANPS 3.1 SADNESS and FEAR scales as representing broader life dimensions being even associated with meaningful pleasant feelings (in absence of their activation) aside from their more typically discussed unpleasant and painful psychological states.

## 2. Methods

### 2.1 Subjects

All subjects were required to be fluent English speakers and be at least 18 years of age. Participants were recruited via the website www.anps-research.com, where participants were provided with insights on their ANPS scores after having filled in the questionnaire. All participants provided informed e-consent and agreed upon the open-science nature of this study (so that anonymous data can be openly shared).

### 2.2 Data cleaning steps

An initial sample of *N* = 863 participants could be collected, who filled in both the ANPS and the Satisfaction with Life Scale. *N* = 46 participants reported to be younger than 18 years and therefore were discarded. This resulted in *n* = 817 participants. Five participants were further discarded because they answered across all ANPS items with answer option 1 (strongly disagree). Further, one person was discarded because this person answered all ANPS items with answer option 4 (slightly agree). Finally, one person was discarded who answered all ANPS items with answer option 6 (strongly agree). The sample size was now *n* = 810. Finally, we discarded participants who stated to be non-native speakers *and* not fluent in English (*n* = 47). The final sample consisted of *n* = 763 participants. From these 425 (55,7%) stated to be English native speakers and 338 (44,3%) not to be English native speakers (but fluent in English). These samples were analyzed independently of each other with the 338 fluent English speakers being used as a hold-out validation sample (in this work we describe the final sample of fluent English speakers also as non-native English speakers). Please note that a recent paper investigated a subsample of the present data in the context of primary emotional systems and attitudes towards AI (Montag et al., 2024).

### 2.3 Sample characteristics

Sample 1 consisted of 425 subjects drawn from a community sample (females: *n* = 237, males: *n* = 188; mean age = 39.80, standard deviation = 16.04). Education level attained was measured on a 6-point scale with 1 = less than high school, 2 = high school graduate, 3 = some college, 4 = bachelor’s degree, 5 = master’s degree, and 6 = doctoral degree. The mean education was 4.17 with a standard deviation of 1.20. Hence, this group was highly educated with the mean being a bachelor’s degree.

Sample 2 consisted of 338 subjects drawn from the same community sample who rated themselves as fluent but not native English speakers (females: *n* = 206, males: *n* = 132, mean age = 32.93, standard deviation = 12.38). Education for the second sample was measured on the same scale as the main sample: mean = 4.09, standard deviation = 1.23. Hence the second sample possessed very similar education levels with the mean being a bachelor’s degree.

### 2.4 Questionnaires

All subjects completed the Affective Neuroscience Personality Scales version 3.1 (ANPS 3.1) (Montag, Elhai, & Davis, 2021; first version of ANPS see here: Davis et al., 2003), and the Satisfaction With Life Scale (SWLS) (Diener et al., 1985). Both assessments were completed in English.

The ANPS 3.1 consisted of 112 items using a 6-point response scale (1 = Strongly Disagree, 2 = Disagree, 3 = Slightly Disagree, 4 = Slightly Agree, 5 = Agree, and 6 = Strongly Agree). The ANPS measures the expression of six primary emotions as defined by Panksepp (1998): SEEKING, ANGER, FEAR, CARE, SADNESS, and PLAY (no LUST scale). Each of these six primary emotion scales includes 14 items with 7 positively worded and 7 negatively worded items. While other experimental items are included in the ANPS 3.1, only the primary emotion scales were used. The names of the primary emotions are written in uppercase to use commonly understood terms and avoid the use of a special vocabulary but at the same time to indicate that the names of each of the primary emotions represent a complex brain system grounded in scientific research.

The SEEKING scale is defined as anticipating positive life outcomes. It has a strong dopaminergic component and is likely foundational for aspects of each of the other primary emotions. The ANGER system is typically aroused when there is a challenge or obstruction to obtaining or trying to retain a valued life resource and, as such is often expressed in “hotheaded” fighting but also reflects the frustrations and irritations of life. FEAR is closely linked to “physical” pain and bodily danger and is often expressed as worry and anxiety regarding such aversive experiences (for discussion of differences between fear and anxiety see Montag, Solms, et al. (2022)). The CARE system at its core is a proto-empathy system that involves CAREing for young offspring that likely explains why females tend to exhibit higher sensitivities for this primary emotion, which often extends as nurturing sympathetic responses to vulnerable others. The SADNESS emotion is closely linked to “psychological” pain, which results from becoming socially separated from loved ones. It is acutely expressed by crying and feelings of sadness and loneliness if reunion with loved ones does not occur. Social PLAY is a uniquely mammalian emotion that is most strongly expressed by children and adolescents. Especially in young children, the need for social play often becomes stronger the longer the period without opportunities to play. While typically associated with “youngsters,” some adults retain more playful capacity than others.

Internal consistencies for sample 1 are as follows: SEEKING: α = .80, FEAR: α = .93, CARE: α = .83, ANGER: α = .86, PLAY: α = .86, SADNESS: α = .86.

Internal consistencies for sample 2 are as follows: SEEKING: α = .78, FEAR: α = .90, CARE: α = .78, ANGER: α = .88, PLAY: α = .86, SADNESS: α = .82.

The SWLS consisted of 5 items rated on a 7-point scale (1 = Strongly Disagree, 2 = Disagree, 3 = Slightly Disagree, 4 = Neither Agree nor Disagree, 5 = Slightly Agree, 6 = Agree, 7 = Strongly Agree). Ed Diener conceived the SWLS as a measure of “global” life satisfaction. He used factor analysis in an attempt to remove elements of positive or negative affect and construct a “narrowly focused [scale] to assess global life satisfaction [that] does not tap related constructs such as positive affect or *loneliness*” [italics added] (Diener et al., 1985) (p. 71).

Internal consistencies for the SWLS are as follows: sample 1: α = .88 and sample 2: α = .86.

### 2.5 Statistical analysis

We present in Tables 1 and 2 the descriptive statistics of the samples including insights into the male and female subsamples. *T*-tests were used further to investigate if male and female subsamples differ in the measures of interest. Further, we present for both samples Pearson correlation analysis to test our hypothesis on ANPS-life satisfaction associations. Finally, regression models were created to get insights into the explained variance of satisfaction with life, when simultaneously considering the primary emotional systems. Please note that the analysis has been conducted with the Jamovi package 2.4.8.0 and SPSS 20.0.0 (SPSS was used for regression analysis only). The data sets underlying this paper are openly shared via the Open Science Framework: https://osf.io/de7zr/. Therefore, the data can be used to run further and own analysis on the data set.

**Table 1.** Descriptive statistics of sample 1 (native) and sample 2 (non-native)

									Skewness		Kurtosis	
	Native	N	Missing	Mean	Median	SD	Minimum	Maximum	Skewness	SE	Kurtosis	SE
SWLS	native	425	0	22.66	23.00	7.363	5.00	35.00	–0.4506	0.118	–0.6327	0.236
	non-native	338	0	23.34	24.00	6.660	5.00	35.00	–0.6206	0.133	–0.2035	0.265
SEEKING	native	425	0	4.51	4.57	0.641	2.50	6.00	–0.3143	0.118	0.1005	0.236
	non-native	338	0	4.45	4.50	0.641	2.21	5.93	–0.4451	0.133	0.3651	0.265
FEAR	native	425	0	3.96	4.07	1.037	1.07	5.93	–0.3490	0.118	–0.5432	0.236
	non-native	338	0	3.96	4.04	0.951	1.36	6.00	–0.2716	0.133	–0.4816	0.265
CARE	native	425	0	4.42	4.50	0.761	1.71	5.93	–0.4453	0.118	0.0919	0.236
	non-native	338	0	4.22	4.21	0.729	1.50	5.86	–0.5814	0.133	0.9499	0.265
ANGER	native	425	0	3.39	3.36	0.819	1.07	5.64	0.0387	0.118	–0.3102	0.236
	non-native	338	0	3.44	3.50	0.924	1.36	5.93	0.1043	0.133	–0.4336	0.265
PLAY	native	425	0	4.29	4.36	0.791	1.79	5.93	–0.5106	0.118	0.0428	0.236
	non-native	338	0	4.08	4.07	0.824	1.43	6.00	–0.3103	0.133	–0.0360	0.265
SADNESS	native	425	0	3.93	4.00	0.847	1.50	5.79	–0.2585	0.118	–0.2628	0.236
	non-native	338	0	3.88	3.86	0.779	2.07	5.93	–0.0549	0.133	–0.1900	0.265

**Table 2.** Descriptive statistics of sample 1 (native) and sample 2 (non-native) for the male (1) and female (2) subsamples

										Skewness		Kurtosis	
	Native	Gender	N	Missing	Mean	Median	SD	Minimum	Maximum	Skewness	SE	Kurtosis	SE
SWLS	native	1	188	0	21.86	23.00	7.846	5.00	35.00	–0.39060	0.177	–0.7886	0.353
		2	237	0	23.29	25.00	6.908	5.00	35.00	–0.45129	0.158	–0.5765	0.315
	non-native	1	132	0	21.96	23.50	7.336	5.00	35.00	–0.55347	0.211	–0.4838	0.419
		2	206	0	24.23	25.00	6.042	7.00	35.00	–0.52873	0.169	–0.3569	0.337
SEEKING	native	1	188	0	4.46	4.50	0.667	2.50	5.93	–0.20619	0.177	–0.0573	0.353
		2	237	0	4.55	4.57	0.618	2.50	6.00	–0.39489	0.158	0.3025	0.315
	non-native	1	132	0	4.43	4.50	0.685	2.21	5.79	–0.51936	0.211	0.3077	0.419
		2	206	0	4.46	4.50	0.614	2.21	5.93	–0.36396	0.169	0.3614	0.337
FEAR	native	1	188	0	3.74	3.86	1.086	1.07	5.93	–0.25019	0.177	–0.6217	0.353
		2	237	0	4.13	4.29	0.964	1.71	5.93	–0.35213	0.158	–0.6081	0.315
	non-native	1	132	0	3.91	4.00	0.972	1.36	5.79	–0.32144	0.211	–0.2653	0.419
		2	206	0	3.99	4.07	0.939	1.57	6.00	–0.23315	0.169	–0.6402	0.337
CARE	native	1	188	0	4.15	4.14	0.784	1.71	5.71	–0.37199	0.177	–0.0496	0.353
		2	237	0	4.63	4.64	0.672	2.29	5.93	–0.33964	0.158	–0.1142	0.315
	non-native	1	132	0	4.04	4.14	0.713	1.50	5.79	–0.73630	0.211	1.5775	0.419
		2	206	0	4.35	4.36	0.715	1.93	5.86	–0.54939	0.169	0.6379	0.337
ANGER	native	1	188	0	3.33	3.36	0.854	1.36	5.64	0.12494	0.177	–0.3108	0.353
		2	237	0	3.44	3.43	0.790	1.07	5.57	–0.01642	0.158	–0.2846	0.315
	non-native	1	132	0	3.57	3.57	0.917	1.36	5.57	–0.04626	0.211	–0.3007	0.419
		2	206	0	3.35	3.46	0.921	1.50	5.93	0.20509	0.169	–0.4220	0.337
PLAY	native	1	188	0	4.25	4.29	0.837	1.79	5.93	–0.47200	0.177	–0.0471	0.353
		2	237	0	4.32	4.43	0.753	1.86	5.79	–0.52751	0.158	0.0901	0.315
	non-native	1	132	0	4.10	4.07	0.867	1.57	5.86	–0.33461	0.211	–0.2232	0.419
		2	206	0	4.06	4.07	0.798	1.43	6.00	–0.30058	0.169	0.1343	0.337
SADNESS	native	1	188	0	3.66	3.57	0.890	1.50	5.71	0.00903	0.177	–0.5188	0.353
		2	237	0	4.15	4.21	0.747	1.86	5.79	–0.31462	0.158	0.1971	0.315
	non-native	1	132	0	3.73	3.79	0.725	2.07	5.93	–0.01998	0.211	0.0832	0.419
		2	206	0	3.97	4.07	0.800	2.14	5.93	–0.13542	0.169	–0.2699	0.337

## 3. Results

In Tables 1 and 2 descriptive statistics are presented for both samples under investigation. As several gender associations appeared, we also investigated associations of interest while controlling for gender.

On our main findings: Despite Diener’s efforts to construct a purely cognitive measure of global life satisfaction, our results suggest that his goal was not fully achieved. While the SWLS seems to be a good measure of global life satisfaction, at a minimum it includes a strong affective component consistent with Panksepp’s primary emotions.

The first look at the data indicated that gender differences existed in both the ANPS 3.1 and the SWLS (see Table 2). Hence, in our main sample of 425 subjects from sample 1, partial correlations were run between the two assessments controlling for gender. These partial correlations between the SWLS and the ANPS scales (see Table 3, left side) showed that with the exception of the CARE scale, the correlations between the two instruments were moderate to strong. Specifically, the SWLS scale’s highest correlations with the ANPS were with the FEAR scale (*r* = –0.512, *p* < .001) and with the ANPS SADNESS scale (*r* = –0.400, *p* < .001). The correlation of the SWLS with ANGER was lower but still significant (*r* = –0.276, *p* < .001). For the positive emotions, correlations with the SWLS, the SEEKING scale correlated moderately (*r* = 0.330, *p* < .001) as did PLAY (*r* = 0.319, *p* < .001), with CARE having a much smaller correlation with SWLS (*r* = 0.118. *p* < .015) but accounting for less than two percent of the variance.

**Table 3.** Correlations between ANPS and *Satisfaction with Life* depicted including ranks (partial correlations controlling for gender are presented); sample 1: *n* = 425 native English speakers; sample 2: *n* = 338 non-native English speakers

ANPS (sample 1)	Satisfaction with life (sample 1)	Rank order (sample 1)	ANPS (sample 2)	Satisfaction with life (sample 2)	Rank order (sample 2)
SEEKING	*r* = 0.33, p < .001	3	SEEKING	*r* = 0.282, *p* < .001	4
ANGER	*r* = –0.276, *p* < .001	5	ANGER	*r* = –0.209, *p* < .001	5
FEAR	*r* = –0.512, *p* < .001	1	FEAR	*r* = –0.454, *p* < .001	1
CARE	*r* = 0.118, *p* = .015	6	CARE	*r* = 0.190, *p* < .001	6
SADNESS	*r* = –0.400, *p* < .001	2	SADNESS	*r* = –0.353, *p* < .001	2
PLAY	*r* = 0.319, *p* < .001	4	PLAY	*r* = 0.290, *p* < .001	3

We ran the same procedure with our hold-out validation sample 2 with “fluent” English-speaking subjects (hence the non-native group - also see Table 3, right side), which confirmed our initial results but with mostly slightly lower correlations, which would be consistent with greater error variance due to reduced language proficiency. In the hold-out sample, the SWLS correlation with ANPS FEAR was *r* = –0.454 (*p* < .001) and the correlation with SADNESS was *r* = –0.353 (*p* < .001). The correlation of the SWLS with ANGER was lower but still statistically significant (*r* = –0.209, *p* < .001). For the positive emotions, the SWLS correlated moderately with SEEKING (*r* = 0.282, *p* < 0.001) as did PLAY *r* = 0.290, *p* < 0.001). In addition, the CARE scale also correlated significantly with the SWLS scale (*r* = 0.190, *p* < .001), but accounted for less than 4 percent of the variance. In both samples, except for the CARE scale, all correlations of the primary emotions with SWLS were highly significant (*p* < .001). Further, the rank order of the ANPS 3.1 scale correlations with the SWLS scale were nearly identical in both samples suggesting a consistent validation pattern reflecting the relative importance of the primary emotions for experiencing high global satisfaction with life (see Table 3, rank information in both samples).

To gain more detailed information of how Panksepp’s primary emotions might influence global satisfaction with life (the present data is cross-sectional), we also correlated the ANPS 3.1 “scale items” with the SWLS scale (see Table 4). At the item level in the main sample, the strongest partial correlations controlling for gender were especially revealing with seven items correlating robustly with SWLS elaborating probable highly specific key predictors of global life satisfaction as measured by the SWLS. As seen from the “rank” column for the main sample, the items with the highest correlations dealt with personal tendencies toward experiences of loneliness, sadness, worrying, and having difficulty relaxing. On the left side of the Table 4 further items are presented for illustrative purposes, which appeared to be of stronger relevance in the hold out sample.

**Table 4.** Highest relevant item level correlations of ANPS 3.1 and Satisfaction with Life in both samples (controlling for gender) sample 1: *n* = 425 native English speakers; sample 2: *n* = 338 non-native English speakers

ANPS (sample 1)	Satisfaction with life (sample 1)	Rank order (sample 1)	ANPS (sample 2)	Satisfaction with life (sample 2)	Rank order (sample 2)
ANPS 002 (FEAR+): People who know me well would say I am an anxious person.	*r* = –0.408, *p* < .001	7	ANPS 002 (FEAR+): People who know me well would say I am an anxious person.	*r* = –0.302, *p* < .001	
ANPS 006 (SADNESS+): I often feel sad.	*r* = –0.487, *p* < .001	2	ANPS 006 (SADNESS+): I often feel sad.	*r* = –0.455, *p* < .001	1
ANPS 018 (FEAR+): I often think of what I should have done after the opportunity has passed.	*r* = –0.328, *p* < .001		ANPS 018 (FEAR+): I often think of what I should have done after the opportunity has passed.	*r* = –0.358, *p* < .001	7
ANPS 030 (SADNESS-): I rarely become sad.	*r* = 0.415, *p* < .001	6	ANPS 030 (SADNESS-): I rarely become sad.	*r* = 0.408, *p* < .001	4
ANPS 038 (SADNESS+): I often feel lonely.	*r* = –0.524, *p* < .001	1	ANPS 038 (SADNESS+): I often feel lonely.	*r* = –0.432, *p* < .001	3
ANPS 050 (FEAR+): I sometimes cannot stop worrying about my problems.	*r* = –0.450, *p* < .001	4	ANPS 050 (FEAR+): I sometimes cannot stop worrying about my problems.	*r* = –0.342, *p* < .001	
ANPS 066 (FEAR+): I often worry about the future.	*r* = –0.379, *p* < .001		ANPS 066 (FEAR+): I often worry about the future.	*r* = –0.381, *p* < .001	6
ANPS 074 (FEAR-): There are very few things that make me anxious.	*r* = 0.434, *p* < .001	5	ANPS 074 (FEAR-): There are very few things that make me anxious.	*r* = 0.323, *p* < .001	
ANPS 090 (FEAR-): I rarely worry about my future.	*r* = 0.370, *p* < .001		ANPS 090 (FEAR-): I rarely worry about my future.	*r* = 0.382, *p* < .001	5
ANPS 098 (FEAR+): I often feel nervous and have difficulty relaxing.	*r* = –0.474, *p* < .001	3	ANPS 098 (FEAR+): I often feel nervous and have difficulty relaxing.	*r* = –0.335, *p* < .001	
ANPS 101 (PLAY+): I see life as being full of opportunities to have fun.	*r* = 0.384, *p* < .001		ANPS 101 (PLAY+): I see life as being full of opportunities to have fun.	*r* = 0.433, *p* < .001	2

**Figure 1.**
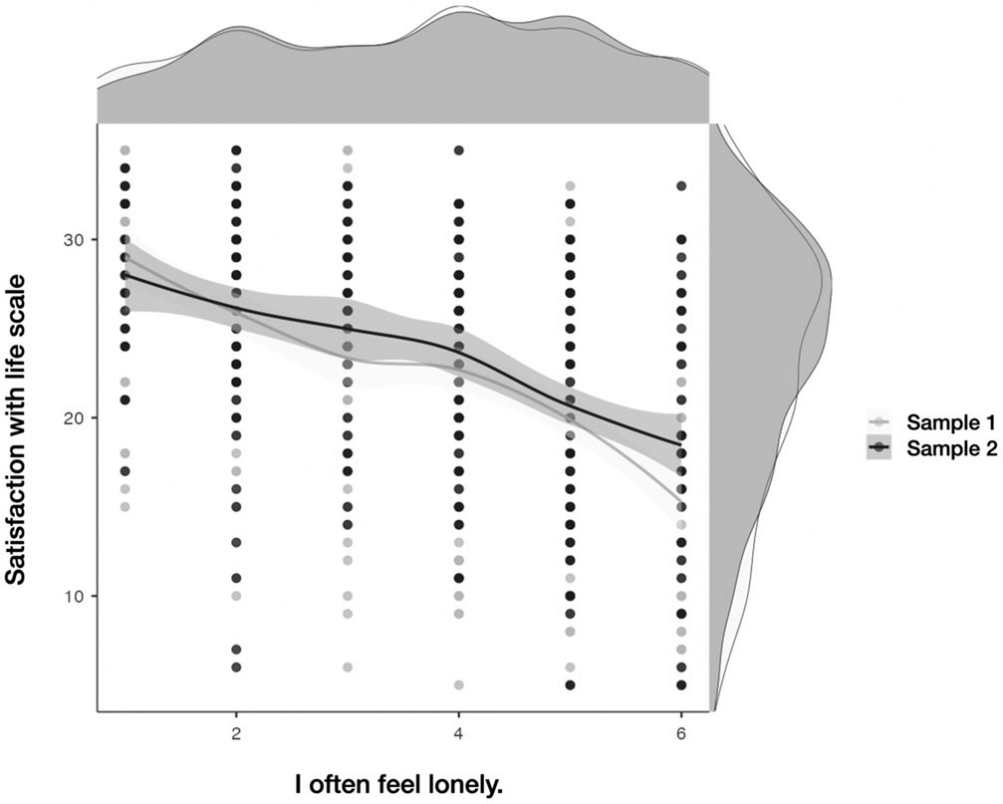
Depiction of one critical SADNESS item to shed light on satisfaction with life (figure produced with the Jamovi package (scatr-tool, regression line and standard errors; presentation-mode: smooth).

Again, the hold-out sample of fluent English speakers largely validated this pattern of affective contributions to global life satisfaction. However, in the hold out validation sample, in addition to the highly ranking ANPS SADNESS items (items 6 and 38; see also Figure 1 depicting associations between item 38 and life satisfaction in both samples), the ANPS PLAY item 101 had the second highest correlation with the SWLS suggesting that frequent PLAY opportunities could contribute more to global life satisfaction than was observed in our first sample of 425 native English speakers. In addition, the three SADNESS items correlated more strongly with the SWLS than any of the FEAR items. However, there were seven ANPS FEAR items that were included in the two lists of the seven highest items correlating with the SWLS across the two samples. So, the item level analysis suggests that while feelings of sadness and loneliness might be the biggest *detractors* from global satisfaction with life, frequently worrying about the future and having difficulty coping with life’s anxieties are also likely strong specific predictors of lower global satisfaction with life. However, “opportunities to have fun” was also included in the top seven correlations for the second sample suggesting it is also a strong element of global satisfaction with life.

So, despite Diener’s best efforts to create a psychometrically pure cognitive scale, the SWLS seems to reflect a strong “affective” evaluation component revealing the importance of the absence of “loneliness” and “sadness,” along with the absence of “worry,” and feeling “nervous” plus frequent “opportunities to have fun” in one’s life. Again, one might ask whether it is possible to make “life judgments” that do not reflect an integration of the quality of experienced life emotions.

To obtain a clearer picture of the overall contribution of Panksepp’s primary emotions on the SWLS, we conducted a regression analysis starting with the two pain-related primary emotions (SADNESS and FEAR) and entered these two scales into the regression equation followed by entering the remaining primary emotions using the forward method with SWLS as the dependent variable. The first model including only the ANPS SADNESS and FEAR scales (*F*_(2,422)_ = 64.414, *p* < .001, *R* = .484, *R* ^2^ = .234) explained 23.4% of the variance associated with the SWLS. Model 2 somewhat surprisingly added the ANPS CARE scale (*F*_(3,421)_ = 58.615, *p* < .001, *R* = .543, *R* ^2^ = .295). Model 3 added the ANPS SEEKING scale (*F*_(4,420)_ = 49.752, *p* < .001, *R* = .567, *R* ^2^ = .321). Finally, Model 4 added the ANPS PLAY scale (*F*_(5,419)_ = 41.579, *p* < .001, *R* = .576, *R* ^2^ = .322) increasing the explained variance to 32.2% shared between the ANPS and SWLS. The ANPS ANGER scale did not reach the *p* = .05 entry requirement.

Running the same regression analysis with the hold-out validation sample using fluent English speakers resulted in only ANPS CARE and SEEKING being added after initially entering the SADNESS and FEAR scales and with PLAY just missing the *p* = .05 entry requirement. Again, the ANPS ANGER scale did not reach the *p* = .05 entry requirement. As expected, the final model using the hold-out sample explained somewhat less variance between the ANPS and SWLS, namely, 29.3%.

## 4. Discussion

Our data provided evidence that ratings of global life satisfaction were significantly associated with a person’s trait SADNESS system and provided evidence that the SWLS assessment likely is an affective evaluation rather than a purely cognitive one. In any case, a partial acknowledgment of an SWLS link to affect came from a review article by Pavot and Diener (1993), which cited correlations of Extraversion and Neuroticism with the SWLS and acknowledged that Extraversion and Neuroticism were characteristics that accounted the long-term stability of SWLS ratings. Further, the correlation with Extraversion was given a more cognitive/behavioral interpretation, namely, “extroverted individuals have more sensitive reward systems” (Pavot & Diener, 1993, p. 168). The correlation with Neuroticism was similarly explained away by saying “individuals who are satisfied with their lives are in general well-adjusted and free from psychopathology” (Diener et al., 1985, p. 73), a statement that seems to assume that psychopathology and psychological adjustment have little to do with emotions or emotional affects. However, in defense of Diener and his colleagues, the original paper (Diener et al., 1985), also suggested that further research should explore the relationship between affect and life satisfaction.

In the already mentioned SWLS review paper (Pavot & Diener, 1993) more attention was given to affect-related measures. For example, they reported correlations between the SWLS and the PANAS positive affect scale (*r* = 0.44) and the negative affect scale (*r* = –.48). They also reported a strong negative correlation between the SWLS and the Beck Depression Inventory (*r* = –.72), which is consistent with our finding of strong negative correlations between the ANPS SADNESS scale as well as ANPS items dealing with high levels of loneliness and sadness.

Pavot and Diener (2008) presented additional evidence that could be used for an affective interpretation of the SWLS. For example, they cited work that showed psychiatric patients had lower SWLS scores than a non-psychiatric control group (Arrindell et al., 2001) and that psychiatric disorder comorbidities further reduced life satisfaction scores (Meyer et al., 2004), which, given the close link between Panksepp’s primary emotions and psychiatric disorders (Montag, Elhai, & Davis, 2021; Panksepp, 2006), underscores the relationship between life satisfaction and emotions and their emotional affects. In this realm, also a work is interesting showing a negative correlation between life satisfaction (using an alternate adolescent scale of life satisfaction) and alcohol and drug abuse in a large study (*n* = 5032) of adolescents (Valois et al., 2001). A related study of middle school students (*n* = 2138) relating violent behavior and presumed high anger levels to reduced life satisfaction is also of interest (Valois et al., 2006).

There have also been three other reports that highlighted “loneliness” as the most significant correlate of low scores on the SWLS (Goodwin et al., 2001; Neto, 1993, 1995). These findings support the high correlation of the ANPS 3.1 SADNESS item 38, “I often feel lonely,” with SWLS (*r* = –.524, *p* < .001) in the main native English-speaking sample and support the strong link between low scores on the SWLS and the high activity of the primary-process SADNESS system as measured by the ANPS 3.1 SADNESS scale. As an exclamation point to these studies, the World Health Organization has recently declared loneliness to be a global health threat.^1^

Further, Pavot and Diener (2008) also cited links between suicide and low life satisfaction with two works (Heisel & Flett, 2004; Moum, 1996) focusing on strong links between the SWLS and suicide ideation. Since, suicide is typically viewed as an escape from pain, which is typically *psychological* pain linked with Panksepp’s SADNESS system more than *physical* pain (Yovell et al., 2016), the association of suicide measures with low global satisfaction with life links low life satisfaction to severe emotional affective SADNESS system distress.

However, a study (Koivumaa-Honkanen et al., 2001) presented an especially compelling story tracking 20 years (1976–1995) of suicide in Finland with a very large sample of Finnish adults (*N* = 29 173). This study used an earlier 4-item life satisfaction scale (LSS) that covered interest in life, happiness vs. sadness, feelings of loneliness, and ease of life. The lowest LSS scores were strongly associated with suicide even after controlling for age, sex, baseline health status, alcohol consumption, smoking status, and physical activity. The Finnish longitudinal study of life satisfaction and suicide along with the previously cited studies of the SWLS and suicide ideation suggest a strong link of low opioid tone to suicide, which was tested by Yoram Yovell and Jaak Panksepp (Yovell et al., 2016). The Yovell and Panksepp research extended Panksepp’s early opioid model linking low-dose morphine to relief from separation distress in canine puppies (Panksepp et al., 1978) by showing that low-doses of the safe opioid buprenorphine resulted in no suicides and a significant reduction of suicide ideation over the four-week trial. However, we suggest that in addition to opioids, more research on the impact of especially oxytocin levels not only on suicide avoidance but on self-confidence and social competence could further expand our understanding of the biological underpinnings of the affects experienced on the low end of the SADNESS/Separation Distress spectrum.

And, what about those who are not sad and lonely, those who are less sensitive to social separation, and who likely have well-functioning opioidergic/oxytocinergic systems and who experience high global satisfaction with life? What can we say more specifically about the pleasant emotional state on the opposite end of the “sad and lonely” side of the SADNESS affective spectrum? Panksepp began exploring this in early writings about the brain’s opioid system using rat social play as a proxy (Panksepp et al., 1985). He and his group reported findings that low doses of morphine “increased” social play in juvenile rats while the opposite effect was observed with the opioid blocker naloxone. He suggested that low doses of morphine create a psychological state of “social comfort,” which promoted social play and which would correspondingly be reduced in the group receiving the morphine blocker naloxone. In short, a rat’s propensity to engage in social play was manipulated by opioid tone, the same factor that was first shown to reduce separation distress (Panksepp et al., 1978).

Further, manipulating opioid levels was shown to influence social dominance outcomes. If two rat pups were matched for weight, gender, and previous play fighting experience, and one rat pup was given a low dose of morphine with the other given a matching dose of naloxone, the rat given morphine would win (Panksepp & Bivens, 2012, p. 371; Panksepp et al., 1985). Panksepp proposed that slightly boosting levels of brain opioids increased a central psychological state of social strength and self-confidence which promoted winning. By contrast, lowering opioid levels likely induced a psychological state associated with social need, insecurity, and social weakness which promoted losing. Again, reducing arousal of the SADNESS/Separation Distress system was associated with a positive psychological state shift associated with social comfort. In writing from a human perspective, Panksepp later wrote “This system has two prominent and opposing facets. In the first instance, arousal of the GRIEF [called SADNESS/Separation Distress in this paper] system makes us feel bereft and miserable. But, when [separation] distress is alleviated … we feel a deep sense of comfort and security … complete and at ease” (Panksepp & Biven, 2012) (p. 316).

In a previous study, we examined the correlations of the ANPS scales with personality-related adjectives and short phrases. Items that correlated highly with SADNESS were “often sad,” “socially insecure,” and “sensitive to rejection.” On the low SADNESS end of the spectrum associated with global life satisfaction and the SWLS were “socially confident,” and “self-assured.” (Davis & Feren, 2015). These findings support the affective continuum associated with the brain SADNESS/Separation Distress system and are consistent with those offered by Jaak Panksepp and his students in the early papers on brain opioids and play. In addition to strong relationships between SADNESS and opioid manipulations resulting in contrasting behaviors and outcomes with rat play research, additional support for a SADNESS system affective continuum comes from Montag et al. (2017), which examined ANPS scales and the Beck Depression Inventory II in a large sample of both psychologically healthy as well as diagnosed depressed inpatients. The results showed a clear bell-shaped distribution continuum of ANPS SADNESS scores with overlapping distributions of the healthy versus depressed groups. These results were replicated in another large population of healthy as well as clinically treated participants showing an overlapping continuum of SADNESS scores across the two groups (Fuchshuber et al., 2019).

## 5. Conclusion

We have parsed the relationship between the SWLS and Neuroticism in our relatively large main and cross-validated samples and shown that in both cases the relationship with Panksepp’s negatively valenced primary emotions is largely limited to FEAR and SADNESS. At the item level, the picture becomes more specific by focusing on low worrying for FEAR and low sadness and low loneliness for SADNESS and seeing life as opportunities to have fun for the PLAY system. The other two positively valenced primary emotions, SEEKING and CARE, system played a smaller role but contributed to predicting 33.2% of the relationship with the SWLS scale and primary emotional systems in the native English-speaking sample. The ANGER brain system exhibited lower relationships with global life satisfaction. The evidence for these findings will likely be strengthened as enhanced corroborative biometric measurements become available, perhaps from objective wearable technologies (see also discussion around digital biomarkers; Montag, Elhai, & Dagum, 2021). As the present work is also hampered by it cross-sectional character, longitudinal studies shedding light on associations between Panksepp’s primary emotional systems and life satisfaction would be much welcomed.

Our data demonstrated a strong relationship between global satisfaction with life and having the capacity to limit sadness and loneliness in one’s life, to limit worrying, and to include opportunities for social fun in one’s life. However, our data is correlational and does not demonstrate causal relationships. Another limitation of this study is that we had no Big Five Conscientiousness scale, which is a Big Five measure with no strong link to Panksepp’s primary emotions (Marengo et al., 2021) and which is likely more closely linked to cognition. However, our data provide weight to the idea that making a global evaluation about how satisfied one is with one’s life is strongly linked to one’s evaluation of affective states, especially FEAR and SADNESS. To paraphrase one of Jaak Panksepp’s queries, can you have a thought or make a life judgment without a feeling?

## Footnotes

1
https://www.who.int/teams/social-determinants-of-health/demographic-change-and-healthy-ageing/social-isolation-and-loneliness.
